# Which factors promote and prohibit successful implementation and normalization of a healthy school lunch program at primary schools in the Netherlands?

**DOI:** 10.1186/s41043-022-00328-4

**Published:** 2022-10-15

**Authors:** Ellen van Kleef, S. Coosje Dijkstra, Jaap Seidell, Monique H. Vingerhoeds, Ilse A. Polet, Gertrude G. Zeinstra

**Affiliations:** 1 Marketing and Consumer Behaviour Group, Wageningen University, Hollandseweg 1, 6706 KN Wageningen, The Netherlands; 2 Faculty of Science, Amsterdam Public Health Research Institute, Vrije Universiteit Amsterdam, De Boelelaan 1085, Amsterdam, The Netherlands; 3 Food, Health and Consumer Research, Wageningen Food and Biobased Research, P.O. Box 17, 6700 AA Wageningen, The Netherlands

**Keywords:** School lunch program, Implementation, Normalisation process theory

## Abstract

**Background:**

A school provided healthy lunch might help to improve the nutritional quality of children’s lunches. However, in the Netherlands, school lunch programs are not common. The aim of this study was to identify factors that promote or inhibit the implementation of a school lunch program at primary schools, from the viewpoint of school professionals.

**Methods:**

A cross-sectional online survey was conducted among 204 primary school professionals. The normalization process theory and its four constructs (i.e. coherence, cognitive participation, collective action, reflective monitoring) were used to develop questions and interpret findings. Descriptive statistics were used for 14 multiple choice questions (yes, no, don’t know) and thematic content analysis for qualitative responses.

**Results:**

Participants had a shared understanding about how a lunch program differed from current practices. Most participants had the same view on the rationale for implementation (coherence), such as equality among children. Sixty percent expected that a healthy school lunch will contribute to healthier eating by the children. Participants showed different degrees of cognitive participation (46% indicated that healthy school lunch is good idea). Commitment depended on their belief whether providing a healthy lunch was part of their responsibility as school and 30% expected a large effect on their daily work (collective action). When appraising school lunch implementation (reflective monitoring), participants’ concerns focused on feasibility and adaptability of a program in their own school.

**Conclusions:**

The introduction of a school lunch program will require substantial effort, although there is considerable support and understanding about potential benefits. The findings point to a number of preconditions for large-scale introduction, including the need for support—both financially and organizationally—bottom-up involvement of teachers, children and parents and freedom to adapt the program.

## Background

School meal programs are considered to be an important way to improve nutritional habits and consequently public health as they reach children of all socio-economic positions and for over a decade of their lives [1]. Most European countries offer a (partly) subsidized school lunch (e.g., Spain, France, Germany and Sweden), although not all children participate in the school lunch. For example, school lunch in Finland is eaten on average by 70–90% of the children [2]. The UK and Portugal have regulations regarding the provision of school lunches [3]. Most countries offer a hot meal as school lunch and have general or more specific food guidelines for school lunches [4, 5]. In several studies, the lunch of children having school meals was healthier compared to home-brought lunches, as the latter contained, on average, more energy, sugar, saturated fat and salt [6, 7]. This was confirmed by two studies at Danish primary schools where packed lunches were substituted by freely provided school meals [8, 9]. In addition, a recent analysis of dietary intake of primary school children in Sweden showed that the school lunch made a positive contribution to children’s diet, with for instance, lunch providing about half of the daily recommended vegetable intake [10].

In the Netherlands, school days are usually Monday through Friday from 8:30 am to approximately 3:00 pm. Some schools close earlier on Wednesdays [11]. The Netherlands has no tradition of serving a school provided lunch at primary schools. Traditionally, the majority of Dutch children at primary schools went home for at least an hour to have lunch, and a small group stayed at school to have a home-packed lunch consisting of sandwiches with toppings (chocolate sprinkles, peanut butter, and cheese). A recent paper based on data of the Dutch National Food Consumption survey showed that 26.5% of the children drank sugar-sweetened beverages during lunch at home and only a very small group ate some vegetables (7.0%) or fruit (6.5%). Children who had their home-packed lunch at school, drank even more sugar-sweetened beverages (55.6%) and had similarly low intakes of vegetables (6.7%) and fruit (9.6%) [12]. A recent Dutch study of 487 home-packed lunch boxes showed comparable low numbers in that 5% contained fruit, 6% vegetables and 19% of all boxes only contained white bread [13].

Recently, there has been a transition at primary schools in the Netherlands from these traditional lunch patterns toward having a home-packed lunch at school. The time for the lunch break at school varies between 30 and 60 min: 15 min of lunch to eat your own sandwiches, and 15–45 min of free play outside after lunch [11]. The low nutritional quality of lunches brought to school coupled with the transition to eating lunch at school more frequently, where there is no lunch program, provided the rationale for the development of a school lunch. Children at three Dutch primary schools received a free sandwich-based healthy school lunch for a 6-month period in the school year 2018–2019 [14]. Children could choose their sandwiches, toppings, vegetables and drinks from a buffet with products in line with a healthy lunch based on the Dutch Nutritional Guidelines [15]. Lunch consumption data was collected at baseline, at 3- and at 6-months as well as 6-months after the intervention). Similarly to positive findings in other countries, the results in the Netherlands showed among others that the percentage of children consuming vegetables at lunch increased and the percentage children consuming sugary sweetened drinks decreased [16].

The potential success of a school lunch to improve children’s eating habits is, to a large degree, dependent upon how successfully it is embedded in routine school practices. Implementing a school lunch in Dutch primary schools can be seen as a drastic change, as people are not used to it, not even in previous generations. It will have organizational and financial consequences that are not obvious at first glance. Consequently, implementation may not be straightforward. Because of ongoing educational and organisational reforms, a tight curriculum, and a high workload, school professionals may be reluctant to accept a school lunch program. In addition, school staff may feel that parents have the primary responsibility of ensuring that children get adequate nutrition [17, 18].

For a school lunch program to be successfully implemented at primary schools in the Netherlands, it is essential to understand the process of how such a new practice can be embedded in the school context with support of the professionals working at these schools. In other words, how can such a new way of having lunch become normalized into everyday primary school practices within a certain period of time? To answer this question, more insight is needed into the obstacles, requirements and enabling factors as perceived by school professionals with regard to implementation.

Because a school lunch implementation can be seen as a complex intervention, normalization process theory has been chosen as the underlying framework [19, 20]. Complex interventions have a higher chance of failure and the theory focuses on change processes to increase the chance of successful implementation [21]. This theory is originally developed for interventions in healthcare and other institutionalized settings. It has been used to aid intervention development and implementation planning as well as evaluating and understanding of complex intervention implementation processes themselves [22, 23, 25]. For example, Reeve and colleagues [24] conducted an online administrated survey among health care professionals to identify enablers and barriers to a new intervention developed to tailor medicine prescription better toward patients. As such, it helps to understand why interventions do, or do not, get embedded in practice.

The theory centres around four key constructs for examining processes around implementation: (1) *coherence* relates to how a new practice or intervention is understood by the people who are going to work with it and whether they grasp the benefits and meaning, (2) *cognitive participation* (engagement) relates to how willing people are to take part in a new practice and how committed they are, (3) *collective action* refers to people’s view on the operational work that needs to be done to enable an intervention to happen, and (4) *reflexive monitoring* refers to the formal and informal appraisal of the benefits and costs of the intervention over time [20].

The aim of the present study is to identify factors that facilitate or hinder the implementation of a school lunch program at primary schools in the Netherlands from the viewpoint of school professionals. For this study, four research questions were defined, related to the four constructs from the normalization process theory:

- How do school professionals perceive, understand and appreciate the school lunch program, e.g., what does it involve compared to the current way of having lunch and why (coherence)?
- Do school professionals see it as their legitimate role to be involved and are they willing to invest time and energy into it (cognitive participation)?
- Are school professionals prepared to make it work in daily practice, and how would this intervene with existing working practices (collective action)?
- How do school professionals assess whether it is worth the effort over time and do they feel that they need some room to adapt the lunch program based on their own experiences (reflective monitoring)?

## Methods

### Design and survey instrument

A cross-sectional study using an online survey was executed to obtain school professionals’ views about a school provided healthy lunch program. The four key constructs of the normalization process theory were used to generate survey questions, inspired by Murray and colleagues [20]. For each theoretical construct, statements were developed resulting in 14 questions (Table 1). Answer possibilities were similar to the approach taken by Reeve and colleagues [24] who presented the following answer categories: (1) Yes, (2) No, (3) Maybe/not sure, followed by a text box called ‘comments’ where participants could enter qualitative responses. To make the concept of the healthy school lunch concrete for all the respondents and ensure a similar starting point, participants watched a short video [26] regarding how such a healthy school lunch could look like, before they answered the questions.

**Table 1:** Items in survey for each theoretical construct

Theoretical component	Items were proceeded by the sentence: The next questions are about a delivered, healthy school lunch
Coherence	It is clear for me how the school lunch differs from the current way of eating lunch at my school
	I believe that children at my school will eat healthier by offering a school lunch
	I believe that a school lunch will contribute to more equality among children at my school
Cognitive participation	I think that offering a school lunch at my school is a good idea
	Offering a school lunch is compatible with the tasks of my school
	I am willing to invest time and energy to set up a school lunch at my school
	I expect that a school lunch—as shown in the video—needs to be adapted in order to start at my school
Collective action	In the research, we learned that a well-organized and healthy school lunch can only be achieved if there is support in terms of staff (for example, an employee who prepares lunch),financing for lunch products and necessary materials (such as a refrigerator). It is also necessary to reserve about half an hour for lunch. When answering the questions below, you can assume a situation in which support and financing has been arranged for your school for a healthy and well-kept school lunch
	I expect that implementation of a school lunch has large consequences for my daily activities at school
	I am fine with extending the school day at my school in order to have enough time (30 min) for a school lunch
	I expect that a school lunch at my school will enhance my work pleasure
	I expect that my colleagues will be enthusiastic about a school lunch at our school
Reflective monitoring	Imagine that the school lunch has been offered at your school for a few months
	I can visualize that the school lunch can become part of the daily routine at my school
	I would like to know the effects that the school lunch has on the children of my school
	I would like to have the freedom to adapt the school lunch—as shown in the video—at my school

### Participants

In the 2020–2021 school year, there were 6131 primary schools in the Netherlands (excluding special primary education) [27]. Through the intervention program ‘The national school breakfast’ we received a file with e-mail addresses of 4570 schools. The recruitment of school professionals was done by sending an invitation email to these email addresses. Of these, 1043 emails were ‘undeliverable’, meaning 3527 schools received an invitation email (77% of initial sample and more than 57% of all Dutch schools). The schools shared the invitation with the relevant school staff (teachers, directors and support staff. In addition, we have also shared the survey link on our social media channels and shared it with several school and health organizations. In combination with the fact that the number of school professionals vary per school, it is impossible to determine the response rate. In the invitation email, it was stressed that the questionnaire could be filled in by directors, teachers and support staff of primary schools. Ethical approval for this study was given by the Social Sciences Ethical Committee of Wageningen University. Data were collected using Qualtrics, an online survey tool.

### Procedure

After providing informed consent on the first page of the survey, participants watched the video of the healthy school lunch [16, 26]. This video (2:01 min) showed recordings of how the healthy school lunch was organized and what it looked like during our previous study at three primary schools in the Netherlands, including the type of foods and drinks offered, the nutritional guidelines applied to the lunch, the way each of the schools served the lunch and how children appreciated the lunch. After watching the video, participants read the following text about the results of the study: ‘Results of the study showed that that children consumed more vegetables and whole wheat bread and less sugar-sweetened beverages compared to a home packed lunch’.

Data were collected in June 2020, after partly reopening the schools during the pandemic. In the information text we stated: ‘we realise that is a special time for schools because of the Corona crisis. When filling in the questionnaire, we ask you to assume that all children are at school for entire and normal school days’. It was also stated: ‘With the term ‘school lunch’ we mean a provided and healthy sandwich-based lunch which is delivered at school, in which children prepare the sandwiches themselves. Each day, there will be 50 g of vegetables for each child. Each week something special will be offered, such as soup, fish, grilled sandwich or a cooked egg’.

Subsequently, participants completed the questions covering the key constructs. Finally, participants completed background questions on type of professional role, type and size of the school, and current school policy with regard to healthy eating.

### Data analysis

Data analysis involved descriptive statistical analysis of the yes/no/not sure answers (absolute numbers, percentages and crosstabs reports) with a qualitative analysis of the text responses provided in the survey. This was done by first reading all text responses by three members of the research team. In joint meetings, a coding list was developed using the NPT concepts of coherence, cognitive participation, collection action and reflective monitoring as a framework with the possibility to include new codes for emerging themes for barriers and facilitators. Next, all text analyses were coded by two researchers and discussed until agreement in case of disagreements.

## Results

### Participants

The sample consisted of 204 participants, with one respondent not providing demographic information. This respondent was maintained in the dataset.

Table 2 shows that about 50% of the participants were teachers and about a third were directors of mainly regular primary schools. The majority (73%) worked at medium-sized schools. About half of the schools were located in villages and almost 20% in large cities. Whereas all Dutch provinces were covered by the survey, the majority of schools were located in the central and southern part of the Netherlands.

**Table 2:** Demographic characteristics of the school survey participants (N = 203 ^a^)

	N (%)
*Position*	
Teacher	97 (47.8)
Teaching assistant	16 (7.9)
Director	64 (31.5)
Other	26 (12.8)
*Education type *^*b*^	
Regular school concept	180 (88.7)
Alternative school concept	23 (11.3)
*School size*	
< 100 pupils	25 (12.3)
100–200 pupils	84 (41.4)
201–300 pupils	65 (32.0)
> 300 pupils	29 (14.3)
*School location*	
Village not adjacent to a city	62 (30.5)
Village adjacent to city	41 (20.2)
City < 30.000 inhabitants	23 (11.3)
City with 30.000–100.000 inhabitants	40 (19.7)
City > 100.000 inhabitants	37 (18.2)
*Provinces*	
Zuid-Holland	42 (20.7)
Noord-Brabant	31 (15.3)
Gelderland	27 (13.3)
Utrecht	26 (12.8)
Other	77 (37.9)
*Currently participating in school food programs*	
Yes	155 (76.5)
Don’t know	11 (5.4)
No	37 (18.2)
*Healthy food policy at school*	
Yes, healthy eating stimulated and maintained	85 (41.9)
Yes, healthy eating stimulated, not obliged	111 (54.7)
No, no policy/rules	5 (2.5)
I don’t know	2 (1.0)
*Healthy School Logo *^*c*^	
Yes	86 (42.4)
Don’t know	32 (15.8)
No	85 (41.9)
*Nutrition certificate (only asked when yes on question above; N* = *86)*	
Yes	27 (31.4)
Don’t know	28 (32.6)
No	31 (36.0)

^a^ One participant did not provide demographic information, but was kept in the dataset

^b^ Indicator of philosophical and ideological vision of schools on education and a child’s learning. Examples of alternative school concepts are Dalton, Jenaplan, Montessori, Vrije school (Waldorf/free school)

^c^ https://www.gezondeschool.nl/

Three quarters (76.5%) of the schools participated in school food programs targeting healthy eating, such as the EU School Fruit program (52.7%), Taste Lessons (36.5%), Properly Fit (‘Lekker Fit’ in Dutch) (10.3%) or other programs (17.2%). Practically all respondents indicated that their school had rules or a policy for healthy eating at school, with 41.9% indicating that this policy was maintained and 54.7% indicating that healthy eating was stimulated, but not obliged. About 40% of the respondents worked at schools that had the Dutch Healthy School Logo, and a third of these had the certificate focussing on healthy nutrition within schools. The Healthy School Logo program supports schools to work integrally and structurally on a healthy lifestyle [28].

### Coherence

**Fig. 1:**
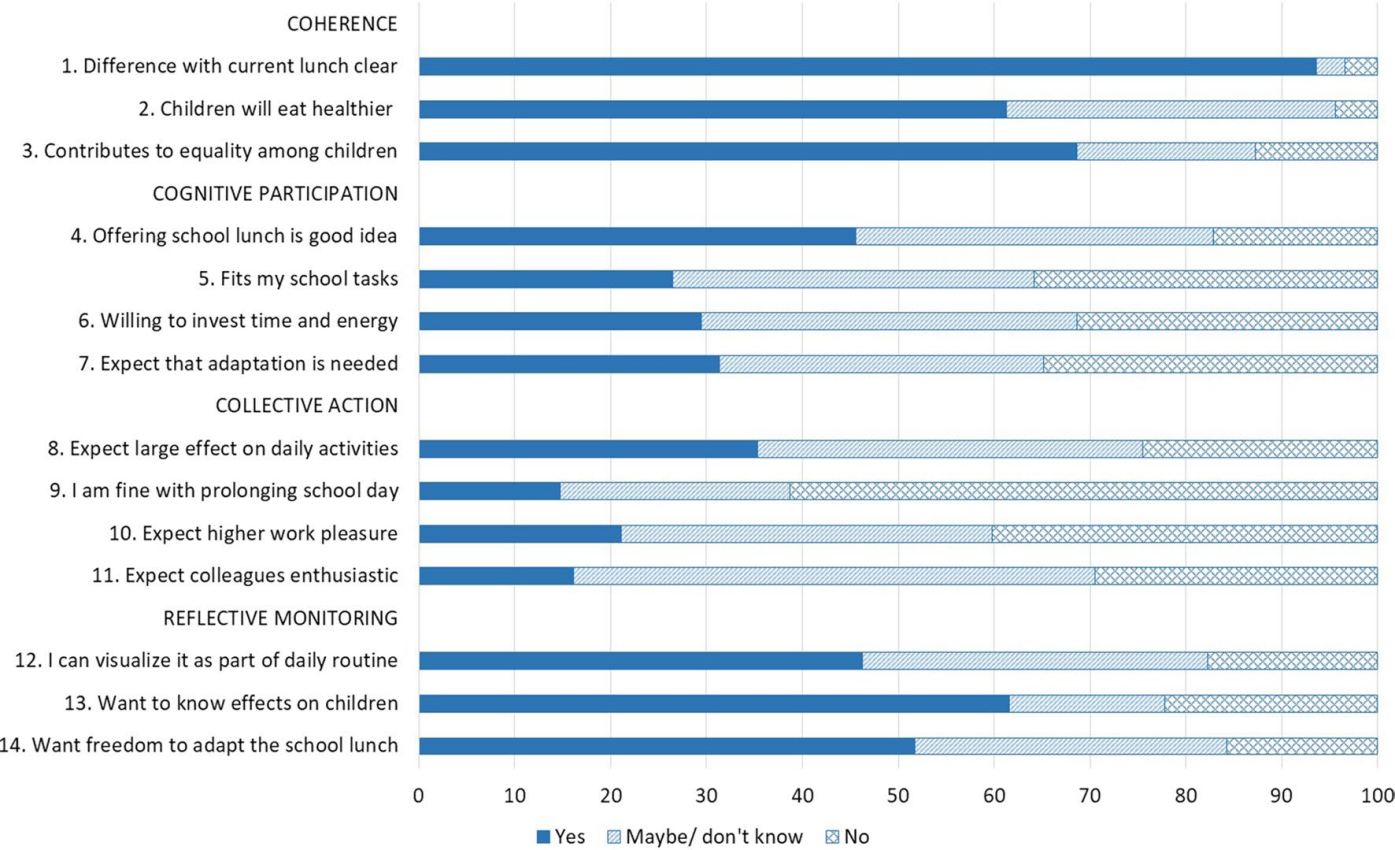
School professionals’ responses given to the 14 items of the survey (N = 204; N = 203 for statements 12, 13, 14)

With regard to coherence, after watching the video, most respondents indicated that it is clear how the healthy school lunch differs from the regular lunch (94%; see Fig. 1). Participants mainly mentioned the difference in lunch offer; the school lunch was seen as healthier and more varied. Some participants emphasized the difference in organization of the lunch, as the common situation is that lunch is brought from home.

Sixty percent of the respondents expected that a healthy school lunch will contribute to healthier eating by the children. Participants cited several explanations for why they thought a school lunch would contribute to a healthier diet. A major reason was that children are exposed to healthier foods that are often not available at their homes. Some participants explained that children now mainly eat what they like, and this is often not healthy. Getting to know different and healthier foods can learn children to appreciate these products: “*They will taste more and eat things that they would otherwise not be offered*”. Current large variety in products and nutritional value of lunch boxes between children was widely mentioned in responses to this statement. Whereas several participants mentioned that children look at each other’s lunch boxes, a few indicated that children hardly look into each other’s lunch, because they are not interested or they are satisfied with what they get themselves. A few participants considered lunch box differences to be small and something to be respected.

About 40% of participants were in doubt or disagreed with the statement regarding the school lunch leading to healthier eating. The majority of explanations given by participants was that children at their school already have a healthy lunch because of school food policies that specify what children can bring for lunch (e.g. “*the large majority of children at my school eats healthy*”). They therefore did not expect any major positive effects of the school lunch. Other participants indicated that there will be little effect for those children who already have a healthy lunch with their own brought products. One participant worried about spoiling parents and children by taking away their responsibility and suggested that it is better to activate parents to purchase and offer healthier foods to their children. Another important concern was that the effect of a healthy school lunch will not contribute to healthier eating at other times of the day (e.g., after school) or even backfires due to parents’ beliefs (“*If it is already ‘healthy’ at school, it is a bit less necessary to eat healthy at home*”). Finally, one participant thought that not all children are willing to try ‘new foods’. This expectation was based on experiences with a school fruit project in which many children refused to eat the offered foods and hence led to a lot of waste.

Almost 70% of the participants agreed with the statement that a school lunch contributes to more equality among children. The reasons stated for this were diverse. It was stated that the school lunch would diminish differences in what children eat at school, as there is typically a huge variety between children in what they eat and drink for lunch. Some packed lunches are healthy, others scanty and unhealthy and there is also a difference in the luxury extras that children bring from home. This often leads to jealousy among children. According to some participants, it also happens that no lunch is brought to school. Participants noted concerns with financial insecurity of parents. For families with smaller incomes, money plays a more important role in the choice of healthy and unhealthy foods. One participant stated: “*A large number of parents live on little or no income and are in debt. Certain parents make choices for cheaper nutrients that are not always healthy*”. Participants highlighted that a school lunch creates the potential for nutrition education on a daily basis. A substantial part of the participants shared this view, summarized in the words of one participant: “*I think children will learn what healthier eating is*”. Some participants indicated to believe that a healthy lunch is important for learning, i.e. being able to perform better in the afternoon.

### Cognitive participation

When looking at the items for cognitive participation, about half of the respondents indicated that the school lunch is a good idea (46%), and almost 30% would be willing to invest time and energy to initiate a school lunch or thought that a school lunch fits their school tasks, with about a third saying ‘no’ to these statements. Almost 40% was unsure on these topics.

Participants thought it was a good idea to encourage healthy eating among children. The reasons mentioned included the importance of making children aware of healthy nutrition, giving them knowledge about what is healthy eating and taking good care of your body (“*Great to make the children aware of healthy nutrition and the importance of taking care of your own body. Especially in these times and age!”*). They mentioned that too often children still bring an unhealthy lunch to school. Some participants also mentioned the social benefits such as creating togetherness through eating together and more variation in the eating pattern. Now, there are also children with too little or without food and that can also be taken care of by a school lunch.

Moreover, responses to the statement ‘offering school lunch is a good idea’ again pointed to arguments related to equality of opportunity for every child in the broadest sense of the word and that a healthy school lunch would promote learning. It was also mentioned that a school lunch could be a relief for the parents since they would not have the stress about preparing lunch for school anymore. Reasons to question a school lunch or to reject the idea of a school lunch mainly concerned the expected increase in workload for teachers. Teachers indicated that they themselves also need a lunch break and the concern is that this lunch break will disappear. Some participants also indicated that the lunch as portrayed seemed organizationally difficult and time-consuming and raised the question of who should take on these tasks in the school, as this cannot be left to volunteers alone. Especially for the younger children, it is assumed to be more difficult to be accomplished within the currently available lunch time. It was stressed by many participants that lunch time should not take away precious education time. The more negative and doubtful responses to this statement emphasized again that the children at their school generally already have a healthy lunch. Some participants also felt there should not be too much patronizing. Responsibility for a healthy diet must lie at home, some of the participants said. It is presumptuous to take responsibility away from them, thinking that parents would not do well. One participant wrote: *“Of course, we want to encourage children to adopt a healthy lifestyle. But parents also have a responsibility. If you give them a school lunch it is very easy for the parents. A little too easy?”.*

Costs were also mentioned and some participant wondered who would pay these costs of a school provided lunch. Other concerns that were put forward were that the school provided lunch would not be suitable for children with allergies or following religious or other dietary rules, and the likelihood of excess leftovers. One participant also indicated that a school lunch appears to lead to restless classrooms, because children have to walk to a buffet to get their food. They indicated that children need a moment of rest during their lunch breaks.

About 30% of the respondents thought that the school lunch—as shown in the video—needed to be adapted for implementation at their school. Adjustments mentioned by participants were about the practical organization in- and outside the classroom, such as finding suitable space and facilities to prepare and store food. One participant stated: *“As a teacher, I have too little time to take on this task in addition to the normal work. However, I see opportunities when a co-worker prepares lunch outside the classroom*.” Adjustments were also considered necessary for younger children, because they may need more help in preparing their own sandwiches. Some participants thought that modifications in the range of available products were necessary, because they do not regard milk and bread as healthy foods and also think that there are parents who think the same. A few indicated they wanted to offer organic food during the lunch.

### Collective action

With regard to collective action, it became clear that the majority (60%) did not want to prolong the school day to have more time for the school lunch, and only 15% said ‘yes’ to this item. Concerns were expressed about the acceptance of a longer school day. The ‘yes’ group participants perceived benefits for the children such as the opportunity to have extra nutrition education and were therefore in favor. Others mentioned that the time-shortening effect of the continuous schedule would then disappear, and this would extend the working day of teachers. This would also lead to challenges for after-school care and sports clubs. A typical lunch break at Dutch primary schools is about 15 min and this short break was also considered to be sufficient by a group of participants. It was stressed that as teachers, they really need the time after school to keep the day-to-day affairs in class and at school in order.

A small group thought that implementing a school lunch would enhance work pleasure (20%), whereas 40% thought this would not be the case. They were uncertain whether colleagues would be enthusiastic about the school lunch, with 54% choosing ‘I don’t know’, 16% indicating ‘yes’, and 29% choosing ‘no’. It was stated by various participants that work pleasure could be reduced by being responsible for too many tasks. At the same time, work pleasure could be increased by the social benefits of eating together every day instead of each child eating from their own lunch box. A few participants indicated that having lunch with the children as a teacher or supervisor is very nice, because it is homely, sometimes even festive and warm, and it ensures more connection and involvement. One participant indicated that when children eat healthier, this will eventually become noticeable: they become healthier, fitter, concentration increases and that ensures more attention and more active posture during gym classes. The opinions of colleagues were expected to be diverse and mixed, for the same reasons mentioned. Some cited resistance to change among colleagues and satisfaction with the status quo.

Whereas 30% expected a large effect on their daily work, 25% did not, and 40% indicated ‘maybe/ do not know’. The time investment for the implementation of a healthy school lunch was seen as significant and it was repeatedly stressed that class time should not be compromised. One participant said it is nice that various tasks related to the school lunch can be done together with the children. In this way they learn a lot, especially when core objectives of education are incorporated into the lunch.

### Reflective monitoring

Concerning reflective monitoring, 46% indicated that they could visualize how the school lunch would become part of the daily routine in their school, whereas 20% could not. The majority of responses were related to feasibility, in the sense that a school lunch would be or could be feasible, when a school chooses to do this. The aspect of time was a theme mentioned both by participants who could visualize it and the ones who could not visualize it. One participant said: *“Yes, I would imagine that two parents would set up a buffet, and the children would pick up their lunch there and eat it in their classroom”, whereas another said: “No, it takes too much education time”*. Other themes that emerged had to do with the added value of a school lunch (“*It fits a healthy lifestyle”*) and that it will take some time to get used to it, but then will become a daily routine (*“If you do it, it will become a daily routine*”).

The majority (62%) would like to know the effects a school lunch would have on the children when implemented in their school. Most responses were related to solely wanting to know the effects (knowledge) or to a kind of cost–benefit analysis of costs (time, effort, and/or money) of the school lunch in relation to the added value. An illustration of the latter is the following quote: “*If you arrange something which takes time and money, then you need to know the actual effects*”. Another participant mentioned that it could be helpful to know the effects with regard to finding funding. Within the themes health and cognition, participants mentioned specific effect outcomes that they wanted to know, such as effects on overweight, healthier food choices, tasting other food, dental health, vigor (health), concentration, working attitude, and learning performance (cognition). From the 40% who did not want to know the effects or were uncertain about this statement, only a few participants gave an explanation. They mainly indicated that they would be able to predict or to know the effects themselves: *“I can estimate that myself*”.

A bit more than half of the participants (52%) wanted to have freedom to adapt the school lunch to their own school. A major theme was “*One size does not fit all*”. It was mentioned that schools differ from each other and that a school lunch concept should fit with the school situation “*No single school is similar to another, so adaptations are always needed*”. One participant mentioned that having a tailor-made approach may increase commitment. Linked to this, the aspect of practical feasibility was mentioned several times, in the sense that adaptations may be needed to ensure feasibility for the teachers. Dietary restrictions and cultural habits formed another important theme underpinning the need for freedom to adapt. One participant very nicely summarized the different responses within this theme: *“It is interesting to connect food to different cultures, religions and countries of origin because food connects. In addition, you have to consider food allergies. I would also like to include the possibilities for regional and seasonal foods”*. Most participants who answered no to this statement referred to the fact that adaptation was not needed, since they would not implement it anyway. A few indicated that adaptations were not needed, or they were unsure about this, as the concept as shown in the video was already good.

## Discussion

Proposing a school lunch program in a country that has no experience with this, means that schools get confronted with a major organizational change. The aim of this research was to understand factors that school professionals perceive to hinder or facilitate a possible introduction of a school lunch program. The normalization process theory was helpful in creating an understanding about how school professionals saw and evaluated a healthy school lunch program in comparison to current practices (coherence), how involved and willing to invest in it they were (cognitive participation), how they would be prepared to put a lunch program into action (collective action) and whether they assessed such program as worth the effort over time and in need for adaptions (reflective monitoring).

Many school professionals see the benefits of a school lunch, but also perceive many uncertainties and issues that still need to be taken care of in the implementation. Professionals working in primary schools in the Netherlands clearly saw how a school lunch programme would be different from current lunch practices, mainly referring to the content of the lunch (healthier and more varied), but also to organisational aspects (i.e. coherence in the normalization theory). A large group (> 60%) did perceive substantial benefits, opportunities and a necessity of a healthy school lunch. It contributes to equality between children and normalizes healthy eating at a time when it is seen as much needed. The 40% of school professionals who were less convinced that a school lunch programme would increase healthiness of the lunch, believed that the quality of the current lunches that children bring home to school are already healthy. This is consistent with findings about breakfast program implementation, where schools sometimes emphasize disadvantages to participation, even if there is evidence for substantial health benefits [29]. In the implementation process, it can be supportive to emphasize long-term benefits to school professionals, in addition to short-term benefits. For example, a recent study shows substantial long-term benefits of school lunch programs in Sweden in reducing socioeconomic inequalities in adulthood [30]. A recent systematic review on universal free school meals also showed that most studies found positive associations with diet quality, food security, and academic performance [31]. Moreover, there is renewed focus on the role of publicly funded school meals in protecting children from the direct effects of poverty and food insecurity [32].

Responses related to cognitive participation in the lunch program revealed important concerns for implementation. Whereas almost half of the sample saw the school lunch as a good idea, about a third stated to be willing to invest time and energy. Teacher workload, costs, organisational aspects were aspects mentioned. Furthermore, the question arose of who has the responsibility for healthy eating among children. Opinions were divided as to whether the school has an important role in this or the parents. Earlier studies already point to this responsibility question and school staff may see parents as primary responsible for adequate nutrition in children [17, 18].

Collective action findings showed how professionals assessed efforts needed to implement a school lunch program in practice. Because there are so many differences in these assessments, the work associated with the collective action needed to implement a healthy school lunch was not generally accepted. Rather, it was hampered by professionals’ concerns on how to achieve a school lunch organizationally, without increasing the workload of the teachers and without reducing the available class time as academic outcomes are their first priority. Struggling with competing priorities is a main challenge that corresponds to findings of studies reporting on factors hindering implementation of healthy lifestyle programs at primary schools [33]. Most school professionals do not want to extend school time, despite the stress they expected on sufficient teaching time when implementing a 30 min lunch break instead of the current 15 min. A solution could be to give the teachers a break by organizing the lunch break by volunteers or pedagogical staff [11].

The ability to adjust the school lunch program was considered important by about half of the participants (i.e. reflective monitoring). This corresponds to findings of Forman and colleagues [34] who studied factors that are important to successful implementation and sustainability of evidence-based interventions in school settings. They interviewed 24 developers of school interventions in the field of mental health. About two-thirds of the developers indicated that they had changed the format or content of the intervention because of problems they encountered when trying to implement and/or sustain the intervention in schools.

Previous research on health program implementation showed that having key people within a school to drive it forward is essential. Leaders within schools need to help prioritize and encourage and guide staff in taking up new programs [33]. In a recent Dutch research project, primary schools were offered daily free healthy lunches and structured physical activity sessions. A process evaluation after a two-year intervention period showed that key conditions to create a positive disruption include enough time, and sufficient bottom-up involvement, external support, team cohesion and coordination. Teachers and parents were involved from the start in the adoption decision and the process of adapting the several changes into the school context [35]. Having the funds available to implement the school lunch next to continuous financing of the meals are therefore critical issues in the implementation. Moreover, the focus should be on each specific school, as each school has their own starting point and process of change [11].

The results of this study should be considered in the light of the following strengths and limitations. A strength of the study lies in its theory driven approach. Moreover, participants watched a video which visualized the practical execution of the school lunch; this enabled a concreter idea of the school lunch than if this needed to be distracted from words. There are also some limitations to this study. Response rate was relatively low and professionals that took part may have been more interested in the topic and thus not representative of the wider population of school professionals in the Netherlands. About 77% of the participants indicated that their school was involved in programs related to healthy eating, which may indicate this response bias. People who are more positive about other programs may also be more likely to be more positive about the school lunch program, so this may give an overly positive picture of the results. It could be an enabler as schools that are familiar with these nutrition or lifestyle programs might have more affinity and knowledge about the meaning of a healthy eating intervention in daily practice. About one third of all schools in the Netherlands are involved in (parts of) the Healthy School Logo Program [36] and around 3000 Dutch primary schools, out of a total approximate amount of 7000, participate in the EU funded school fruit program [37]. For schools that are less interested in healthy eating, it may take more effort to involve them in healthy eating initiatives. Other limitations of our methodology were that not all participants gave an explanation to their responses. This may be due to fatigue in providing answers or due to the fact they had similar explanations for multiple questions, making them less willing to explain their answers fully. The data collection took place during the pandemic. Although this was addressed in the instructions, we cannot exclude that it has influenced participants' answers.

Offering a healthy school lunch at school is one way of supporting a healthy lunch for children. Having a policy about what is brought to school would be another option to support a healthy school lunch, which may sound easier to implement for teachers at first sight. A recent study showed that having a 5-day-fruit-and-vegetable policy for the morning break at Dutch primary schools enhanced children’s fruit and vegetable consumption and was perceived as a feasible strategy [38]. However, it is important to realize that a lunch is a much more complex situation. Whereas it may be relatively easy for teachers to monitor whether children bring fruit and vegetables to school, this is not so easy for a healthy lunch. Whether children bring low fat or high fat versions of cheese or margarine, is difficult to know on the eye. The same is true for preserves (jam) where reduced sugar versions look similar to regular sugar versions and some brown colored breads may be made of white flour (low in fiber). This underpins the importance of the provision of healthy lunch products to the children and having sufficient support for teachers to realize this in practice, as implementation challenges are substantial. To motivate school staff, it is important to show progress results regularly, although currently long-term health effects on children are not yet discernible and require monitoring of results over time. Having the flexibility and autonomy over how to make an intervention fit the specific school context has been shown to increase acceptance and ownership [33]. Giving children a role in the implementation may help to engage them and model healthy eating behaviors to their peers [33]. Recent studies in the Netherlands showed that parents are sympathetic to a school-provided lunch, even when they are not familiar with it [39, 40]. It is important to understand their perspective and involve them in the implementation process.

To conclude, the introduction of a school lunch program in the Netherlands will require substantial effort, although our study showed that there is considerable support and understanding about potential benefits of a school lunch program. The findings further point to a number of preconditions for the large-scale introduction of a school lunch program, including the need for appropriate support—both financially and organisationally—bottom-up involvement of teachers, children and parents and the freedom to adapt the program to their own school situation when needed. The World Health Organization states that a life-course approach should be the starting point for the prevention of chronic diseases such as obesity [41]. This means that cohesive preventive measures must be taken throughout all life stages. A healthy school lunch for every child offers equal opportunities for a healthier life [30].

## Data Availability

The datasets generated and analysed during the current study are not publicly available due to privacy reasons but are available from the corresponding author on reasonable request.
